# Treatment of canine generalized demodicosis associated with hyperadrenocorticism with spot-on moxidectin and imidacloprid

**DOI:** 10.1186/1751-0147-55-40

**Published:** 2013-05-10

**Authors:** Hui-Pi Huang, Yu-Hsin Lien

**Affiliations:** 1 Institute of Veterinary Clinical Science, Veterinary School, National Taiwan University, Roosevelt Road, Taipei 106, Taiwan; 2 Azu Clinic for Animals, KinShan South Road 100, Taipei, Taiwan

**Keywords:** *Demodex canis*, Demodicosis, Dog, Hyperadrenocorticism, Moxidectin

## Abstract

**Background:**

Canine generalized demodicosis associated with hyperadrenocorticism is often problematic and might be intractable. The aim of this study was to report the efficacy of a weekly application of spot-on moxidectin/imidacloprid in dogs with hyperadrenocorticism and secondary generalized demodicosis.

**Methods:**

Dogs with hyperadrenocorticism and secondary generalized demodicosis were included. The condition of hyperadrenocorticism was treated and stabilized with trilostane before and throughout the study period in all dogs.

**Results:**

Average total live adult mite counts before treatment and after four, eight and 12 weeks of spot-on moxidectin/imidacloprid (2.5/10 mg/kg) applications were 20.1 ± 6.3 (range, 13**–**33), 0.5 ± 0.7 (range, 0**–**2; 6/11 were negative), 0.2 ± 0.4 (range, 0**–**1; 9/11 were negative), 0.2 ± 0.4 (range, 0**–**1; 9/11 were negative) and 0.1 ± 0.3 (range, 0**–**1; 10/11 were negative) respectively; this difference was significant (*P* < 0.001). Ten of 11 dogs (90.1%) achieved clinical remission, as demonstrated by the absence of demodectic mites at any life stage at monthly scrapings for eight consecutive weeks, and maintained remission throughout the 12-month follow-up period.

**Conclusion:**

The weekly application of spot-on moxidectin/imidacloprid appeared to be effective and safe against generalized adult onset canine demodicosis associated with hyperadrenocorticism.

## Background

Demodicosis is a relatively common canine skin disease with localized and generalized (juvenile- and adult-onset) forms [1]. Adult-onset canine generalized demodicosis is a multifactorial disease with an etiology including genetic factors and suppressed T-cell immunity [2]. Its development is also associated with immunosuppressive treatments, neoplasia and medical conditions characterized by metabolic changes, such as hyperadrenocorticism, hypothyroidism and diabetes mellitus [3].

Among evaluated therapeutic protocols for canine generalized demodicosis, oral ivermectin has been used most widely worldwide. Milbemycin oxime, also a macrocyclic lactone, has been commonly used in refractory cases [4]. The efficacy of both of these antiparasitic agents is associated with higher dosage and more frequent administration [5-8]. A spot-on formulation of moxidectin, another macrocyclic lactone, is licensed for the treatment of demodicosis in many countries [9] and has recently been registered in the authors’ country of practice.

Hyperadrenocorticism (HAC) is a common canine endocrine disorder characterized by chronically elevated circulating concentrations of the steroid hormones produced by the adrenal cortex [10]. Immunosuppression is a common complication associated with long-term hyperadrenocorticism, which renders dogs prone to the development of secondary demodicosis and is often problematic and might be intractable [3].

The purpose of this study was to evaluate the efficacy of spot-on moxidectin/imidacloprid in dogs with hyperadrenocorticism and associated generalized demodicosis.

## Methods

### Case selection

Client-owned dogs with hyperadrenocorticism and secondary generalized demodicosis, initially not achieving parasitic remission after oral ivermectin (Ivomec^®^; Merial Limited, GA, USA; 0.5 mg/kg, once daily for at least five weeks) in combination with trilostane (Vetoryl^®^; Dechra Pharmaceuticals PLC, Stoke-on-Trent, UK), but had reached maintained clinical and microscopic remission with oral milbemycin oxime (Milbemycin A; Daiichi Sankyo Co., Ltd., Japan; 0.5 mg/kg, once daily for at least four weeks), but later relapsed after milbemycine oxime discontinuation were included. Informed consent was obtained from all dog owners prior to inclusion in the study.

### Diagnosis of generalized demodicosis

Generalized demodicosis was defined as demodicosis affecting more than four small areas (<100 cm^2^) on the body, at least one area > 100 cm^2^, or at least one paw. Four skin scrapings, from four affected areas, were taken in each dog exhibiting at least four lesional areas. The diagnosis of demodicosis was based on the finding of more than three live adult *Demodex canis* mites from at least three of four scrapings, or from lesions > 100 cm^2^ or from a lesional paw [11].

### Diagnosis and stabilization of hyperadrenocorticism

Diagnosis of hyperadrenocorticism had been based on clinical signs (such as polydipsia, polyuria, polyphagia, decreased activity level, panting, a pot-bellied appearance, excessive hair loss or alopecia in general, chronic pyoderma) and the results of routine serum biochemical analyses (such as elevation of hepatic enzyme activities), affirmative adrenocorticotropic hormone stimulation test results and adrenal ultrasonographic images [12]. Stabilized hyperadrenocorticism was defined as alleviation of clinical signs (polydipsia and/or polyuria, activity level and panting) and reduced excretion of post-ACTH cortisol within the therapeutic range (< 10 μg/dL) after four weeks of trilostane treatment.

### Treatment protocol of spot-on moxidectin/imidacloprid

A spot-on formulation of 10% imidacloprid and 2.5% moxidectin (Advocate^®^, 0.1 ml/kg; Bayer HealthCare AG, Leverkusen, Germany) was applied to each dog once weekly for 12 weeks.

All dogs were evaluated every four weeks for 12 weeks: four skin scrapings were taken from four initially affected areas and routine blood tests were performed every four weeks for 12 weeks. Side effects were recorded during the study period.

In this study, the efficacy of spot-on moxidectin/imidacloprid was defined as the absence of *Demodex canis* mites in any life stage at monthly scrapings for eight consecutive weeks. These dogs received no other treatment against ectoparasites during the study period.

Concomitant trilostane as the treatment for hyperadrenocorticism was carried on at unchanged dosage in all dogs throughout the study period.

### Follow up

After withdrawal of spot-on moxidectin/imidacloprid, physical examination, routine blood tests and skin scrapings were performed in all dogs at intervals four to eight weeks over a period of 12 months. The dogs received no treatment against ectoparasites during the period of follow up for 12 months after discontinuation of moxidectin/imidacloprid.

### Statistical analyses

Total numbers of adult mites recovered from skin scrapings taken before treatment and after four, eight and 12 weeks of treatment were compared by one-way analysis of variance (ANOVA). Parameters of routine blood tests taken before treatment and after 12 weeks of treatment were also compared by one-way ANOVA. The Kolmogorov–Smirnov test was used to assess the normality of distribution of numeric and continuous data. One-way ANOVA was used compare normally distributed variables between groups, and the Kruskal–Wallis test was used to compare non-normally distributed variables. All statistical analyses were performed with the SPSS software (version 13.0; SPSS Inc., Chicago, IL, USA). Continuous data are presented as means ± standard deviations. Statistical significance was set at *P* ≤ 0.05.

## Results

Eleven dogs fulfilled the inclusion criteria and were included in this study (Table 1).

**Table 1:** Breed, sex age, body weight, duration of ivermectin therapy prior to and after concomitant trilostane, duration of milbemycin oxime therapy, clinical signs associated with generalized demodicosis (GD) after withdrawal of milbemycin oxime for eight weeks, clinical signs associated with hyperadrenocorticism (HAC) and trilostane dosage of 11 dogs

**No**	**Case**	**Duration of ivermectin therapy prior to/after trilostane (week)**	**Duration of milbemycin oxime therapy (week)**	**Clinical lesions associated with GD after withdrawal of milbemycin oxime (week of withdrawal)**	**Clinical signs associated with HAC and dosage of trilostane**
1	14 y, M, Pomeranian (2.8 kg)	9/6	5	Generalized erythema and pyoderma mainly on both flanks (10)	Polydipsia, polyphagia, polyuria, decreased activity, a potbellied appearance, muscular wasting, localized alopecia and chronic pyoderma (franks);
					2.1 mg/kg/day
2	12 y, FS Yorkshire terrier (2.6 kg)	10/7	5	Generalized erythema and deep pyoderma mainly on back (10)	Polydipsia, polyuria, decreased activity, a potbellied appearance, muscular wasting, generalized alopecia and chronic pyoderma;
					1.9 mg/kg/day
3	12.5 y, FS Maltese terrier (3.8 kg)	8/6	5	Generalized erythema and deep pododermatitis mainly on face and lateral thighs (8)	Polydipsia, polyphagia, polyuria, decreased activity, panting, a potbellied appearance, muscular wasting, generalized alopecia and chronic pyoderma, calcinosis cutis;
					2.3 mg/kg/day
4	11 y, FS Chihuahua (1.8 kg)	9/6	7	Generalized erythema and deep pododermatitis (Front two paws) (8)	Polydipsia, polyphagia, polyuria, decreased activity, panting, a potbellied appearance, muscular wasting, generalized alopecia and chronic pyoderma in general, calcinosis cutis;
					3.3/mg/kg/day
5	13 y FS Maltese terrier (5.1 kg)	6/6	7	Generalized erythema and pyoderma and deep pododermatitis (four paws) (8)	Polydipsia, polyphagia, polyuria, decreased activity, panting, a potbellied appearance, muscular wasting, generalized alopecia and chronic pyoderma in general, concurrent pyonephrosis (one kidney left);
					2.3 mg/kg/day
6	10 y, M Shih Tzu (6.1 kg)	8/6	5	Generalized erythema and pyoderma and deep pododermatitis (four paws) (9)	Polydipsia, polyuria, decreased activity, panting, a potbellied appearance, generalized alopecia and chronic pyoderma;
					2.0 mg/kg/day
7	9 y, MC Shih Tzu (8 kg)	8/6	5	Generalized erythema and deep pododermatitis (front two paws) (8)	Polydipsia, polyphagia, polyuria, panting, a potbellied appearance, muscular wasting, localized alopecia and chronic pyoderma (frank);
					1.9 mg/kg/day
8	13 y, MC Yorkshire terrier (5 kg)	10/7	5	Generalized erythema and pyoderma mainly on back (9)	Polydipsia, polyphagia, polyuria, decreased activity, a potbellied appearance, muscular wasting, generalized alopecia and chronic pyoderma;
					1.7 mg/kg/day
9	11 y, FS Maltese terrier (11.5 kg)	6/6	5	Generalized erythema and pyoderma mainly on ventral chest (8)	Polydipsia, polyphagia, polyuria, decreased activity, panting, a potbellied appearance, muscular wasting, generalized alopecia and chronic pyoderma;
					1.7/mg/kg/day
10	12.5 y, FS Maltese terrier (4.5 kg)	6/6	7	Generalized erythema and deep pyoderma mainly on back (8)	Polydipsia, polyuria, decreased activity, panting, a potbellied appearance, muscular wasting, generalized alopecia, chronic pyoderma and concurrent DM;
					1.9 mg/kg/day
11	15 y, FS Shih Tzu (6.2 kg)	9/6	5	Generalized erythema and pyodermaand deep pododermatitis (two front paws) (8)	Polydipsia, polyuria, decreased activity, panting, a potbellied appearance, muscular wasting in both thighs, generalized alopecia and pyoderma;
					1.8 mg/kg/day
		Period of Ivermectin before/after concomitant trilostane treatment Mean: 8.1/6.2 weeks Median: 8/6 weeks	Period of Milbemycin oxime after concomitant trilostane treatment Mean: 5.5 weeks Median: 5 weeks	Period of non-mitidal treatment after Milbemycin oxime withdrawn Mean: 8.5 weeks Median: 8 weeks	

DM: diabetes mellitus, FS: spayed female, M: male, MC: castrated male, PD: polydipsia, PP: polyphagia, PU: polyuria, y: years.

### Effect of spot-on moxidectin/imidacloprid on clinical signs

Improvement generally became evident in 10 of 11 dogs within four weeks after the start of treatment, with decreased severity of cutaneous inflammation of affected areas. Clinical improvement was evident in all dogs within eight weeks after the start of treatment, including alleviation of inflammation and hair re-growth on the affected areas/dorsal paws.

### Effect of spot-on moxidectin/imidacloprid on live adult mite counts

The average total live adult mite counts before treatment and after four, eight and 12 weeks of spot-on moxidectin/imidacloprid applications were 20.1 ± 6.3, 0.5 ± 0.7 (6/11 were negative), 0.2 ± 0.4 (9/11 were negative) and 0.1 ± 0.3 (10/11 were negative), respectively; this difference was significant (*P* < 0.001; Table 2).

**Table 2:** Mean number of Demodex mites in skin scrapings pre-trilostane treatment, pre- and during spot-on moxidectin/imidacloprid (M/I)

**No**	**Case**	**Total live adult mite count**				
		**Prior to trilostane**	**Pre spot- on M/I**	**After 4 weeks of M/I**	**After 8 weeks of M/I**	**After 12 weeks of M/I**
1	14 y, M, Pomeranian (2.8 kg)	17	15	1	0	0
2	12 y, FS Yorkshire terrier (2.6 kg)	26	28	1	1	0
3	12.5 y, FS Maltese terrier (3.8 kg)	20	18	0	0	0
4	11 y, FS Chihuahua (1.8 kg)	27	25	0	0	0
5	13 y FS Maltese terrier (5.1 kg)	30	33	2	1	1
6	10 y, M Shih Tzu (6.1 kg)	13	17	0	0	0
7	9 y, MC Shih Tzu (8 kg)	17	14	0	0	0
8	13 y, MC Yorkshire terrier (5 kg)	19	18	0	0	0
9	11 y, FS Maltese terrier (11.5 kg)	20	22	1	0	0
10	12.5 y, FS Maltese terrier (4.5 kg)	15	13	0	0	0
11	15 y, FS Shih Tzu (6.2 kg)	16	18	1	0	0

### Side effects of spot-on moxidectin/imidacloprid

The owners of the dogs reported no side effects during the treatment period. Among routine blood test parameters, only pre- and post-treatment white blood cell counts differed significantly (*P* < 0.001; Table 3).

**Table 3:** Results (± standard deviation, range) of routine blood tests performed before and after 12 weeks of treatment with spot-on moxidectin/imidacloprid in 11 dogs with hyperadrenocorticism and generalized demodicosis

**Parameter**	**Reference range**	**Before treatment**	**After 12 weeks of treatment**
Hemoglobin (g/dl)	11.5–18	15.2 ± 1.0	15.2 ± 1.0
		(13.8–16.7)	(13.7–16.7)
Packed cell volume (%)	35–52	42.7 ± 2.6	41.8 ± 3.0
		(39–47)	(38–48)
Red blood cells (× 10^6^/μl)	5.2–7.5	6.1 ± 0.4 (5.5–6.9)	6.0 ± 0.5
			(5.6–7.1)
White blood cells (/μl)	6000–15,500	14,718 ± 1549	11,963 ± 1601^*^
		(12,400–17,600)	(9700–14,200)
Albumin (g/dl)	2.6–3.7	2.9 ± 0.2	2.9 ± 0.2
		(2.6–3.2)	(2.6–3.1)
Alkaline phosphatase (U/l)	27–219	268.5 ± 194.8	244.5 ± 150.0
		(90–752)	(112–630)
Alanine aminotransferase (U/l)	20–123	98.6 ± 33.0 (67–170)	94.6 ± 22.2
			(53–130)
Blood urea nitrogen (mg/dl)	7–27	24.0 ± 5.6	24.5 ± 5.4
		(15–31)	(16–32)
Creatinine (mg/dl)	0.5–1.5	1.4 ± 0.2	1.3 ± 0.2
		(1.1–1.6)	(1.0–1.7)
Glucose (mg/dl)	82–121	108.0 ± 8.3	108.2 ± 11.1
		(97–119)	(90–121)
Globulin (g/dl)	2.8–4.8	4.2 ± 0.3	4.1 ± 0.2
		(3.8–4.6)	(3.8–4.5)

^*^P < 0.05 *vs*. pretreatment.

### Follow up

Ten of 11 dogs were free from clinical lesions associated with generalized demodicosis and maintained microscopic remission over 12 months after withdrawal of spot-on moxidectin/imidacloprid. One dog never reached microscopic remission after 12 months of follow-up and multiple skin scrapings revealed one to three live adult mites throughout the period of following 12 months. Mild alopecia and pyoderma on the dorsal paws remained. However the clinical improvement was appreciated by the owner. The dogs received no other treatment against ectoparasite during the period of follow up for 12 months after discontinuation of moxidectin/imidacloprid.

## Discussion

The estimated efficacy of spot-on moxidectin/imidacloprid treatment was 90.1% over the 12-month follow-up period, which exceeded previously reports of 15–87% for spot-on moxidectin/imidacloprid treatment of canine generalized demodicosis [11,13]. The higher efficacy may be associated with the frequency of application, as in this study the drug was administered once weekly, whereas in the previous reports a two- to four-week interval was used.

The high success rate of treatment might furthermore be associated with the susceptibility of *Demodex canis* mites to the new moxidectin/imidacloprid preparation, which was introduced recently in our veterinary market. Non-exposure to the new moxidectin/imidacloprid preparation might explain a high susceptibility in the demodectic mites. Together with a more frequent application, a high susceptibility of Demodex mites to the new preparation likely can be the explanation of the short intervals between treatment initiation and the first negative skin scrapings (8 weeks in 9/11 dogs) and clinical remission (12 weeks in 10/11 dogs).

The dogs in this study had been treated with other macrocyclic lactones, ivermectin and milbemycin oxime, prior to inclusion with remission achieved. Relapse of generalized demodicosis was presented after discontinuation of milbemycin oxime treatment, prior to inclusion in the study. In this study, the treatment-free period of milbemycin oxim had though not been longer than approximately two months. Although the efficacy of macrocyclic lactones may be variable in treating demodicosis, the mechanism of macrocyclic lactones is similarly. The short treatment-free period of milbemycin oxime might also contribute to the fast resolution of the disease after initiation of the moxidectin/imidacloprid treatment.

All dogs in our series had generalized demodicosis associated with hyperadrenocorticism, a metabolic disorder characterized by multisystemic complications affecting the cardiovascular, digestive (hepatic and pancreatic), urogenital, muscular and/or cutaneous systems. Long-term excessive cortisol concentrations may induce immunosuppression and predispose dogs to generalized demodicosis [3,10]. In this study, 12 consecutive weekly applications of spot-on moxidectin/imidacloprid were well tolerated by these metabolism-compromised dogs, with no reported side effect and no abnormality detected by routine blood tests.

Side effects of topical moxidectin, such as erythema and scaling at the administration site are rarely reported [14], but ataxia, lethargy, loss of appetite and vomiting have been reported in dogs treated with oral moxidectin [15]. The major adverse effects of systemic macrocyclic lactones are neurotoxic, including lethargy, hypersalivation, ataxia, coma, mydriasis, clinical blindness and seizure. Certain breeds of dogs (e.g., standard and miniature Australian shepherds, border collies, collies, German shepherds, longhaired whippets, Old English and Shetland sheepdogs and silken windhounds) commonly display a mutation of the multiple drug resistance gene (MDR1), which may cause defective P-glycoprotein function and increase susceptibility to systemic macrocyclic lactones, including ivermectin and milbemycin, through the accumulation of relatively high drug concentrations in the central nervous system, even when relatively low doses are administered [16]. Adverse effects of ivermectin are uncommon in other breeds and include the neurotoxic effects listed above [5,6]. Milbemycin toxicity is rare and usually non-severe in dogs, including lethargy, vomiting, stupor, ataxia and trembling [8,17]. Thus, milbemycin and spot-on moxidectin are alternative treatments in dogs that are sensitive to ivermectin.

In our cases, clinical and microscopic remission had been maintained with sustained milbemycin. Relapse of demodicosis occurred when milbemycin was withdrawn. A complete remission could possibly have been achieved if milbemycin oxim treatment had been continued. Treatment regimens including milbemycin are expensive and often to be needed life-long. In terms of financial concerns, spot-on moxidectin were more cost effective in these dogs.

One dog (No. 5) in our series never reached microscopic remission, but improved clinically. Although her hyperadrenocorticism was stabilized throughout the study period, her general condition was complicated by chronic unilateral (left) pyonephrosis, which may have resulted in the failure to achieve remission.

Higher concentration of ivermectin and milbemycin seem to be associated with higher efficacy in the treatment of canine generalized demodicosis [5-8]. Similarly, the efficacy of weekly spot-on moxidectin/imidacloprid applications in this study was higher than those of biweekly or monthly regimens. Further evaluation of the efficacy and toxicity of this treatment at higher doses or shorter application intervals is warranted.

Our results demonstrate that the weekly application of spot-on moxidectin/imidacloprid was effective in the treatment of canine generalized demodicosis secondary to hyperadrenocorticism. Twelve consecutive weekly applications of this treatment were well tolerated by these metabolism-compromised dogs, with no side effect observed and no abnormality detected by routine blood tests.

## Competing interests

The authors declare that they have no competing interests.

## Authors’ contributions

HPH participated in the designs of the study, carried out the recruitment of cases (dogs with demodicosis) and performed the statistical analysis and the manuscript writing. YHL participated in the designs of the study and carried out the recruitment of cases (dogs with hyperadrenocorticism). She also drafted the manuscript. Both authors read and approved the final manuscript.
